# Auditory Processing Assessment in older people with no report of hearing disability

**DOI:** 10.1016/S1808-8694(15)30151-8

**Published:** 2015-10-18

**Authors:** Maura Ligia Sanchez, Flavio Barbosa Nunes, Flavia Barros, Mauricio Malavasi Ganança, Heloisa Helena Caovilla

**Affiliations:** a Speech and Hearing Therapist, MSc. Department of Otorhinolaryngology/Head and Neck Surgery - Federal University of São Paulo Medical School (UNIFESP-EPM); b Otorhinolaryngologist. PhD student - Surgery Department - University of Minas Gerais Medical School (HC-UFMG); c Speech and Hearing Therapist, MSc. Department of Otorhinolaryngology/Head and Neck Surgery - Federal University of São Paulo Medical School (UNIFESP-EPM); d Full Professor of Otorhinolaryngology - Department of Otorhinolaryngology/Head and Neck Surgery - Federal University of São Paulo Medical School (UNIFESP-EPM); e Associate Professor of Neurotology - Department of Otorhinolaryngology/Head and Neck Surgery - Federal University of São Paulo Medical School (UNIFESP-EPM); f Disciplina de Otoneurologia da Universidade Federal de São Paulo - Escola Paulista de Medicina (UNIFESP-EPM)

**Keywords:** central auditory diseases, elderly, auditory perceptual disorders, auditory pathways

## Abstract

In the elderly, the results of central auditory pathways behavioral assessments are considered to be difficult to read because of the possible interference of peripheral auditory pathway involvement. **Aim**: Assess the efficacy of the central auditory function in elderly patients who do not complain of hearing. **Materials and Methods**: Case study involving 40 individuals within the age range of 60 to 75 years. The patients underwent auditory processing evaluation based on anamnesis, otorhinolaryngological exam, threshold tonal audiometry, speech recognition threshold, speech recognition index, immittance measures, stapes reflex study, synthetic phrases identification test with ipsilateral competitive message, frequency pattern test and alternate twin-syllable test through dichotic task; age range and hearing loss influenced results from the phrases identification with ipsilateral competitive message. Percentages of right answers below normal standards were seen in the three tests that assessed the central auditory functions. **Conclusion**: Elderly individuals who did not complain of hearing presented relevant prevalence of signs of central auditory function inefficiencies.

## INTRODUCTION

Some elderly have difficulties to understand speech, that do not match the peripheral auditory disorder they have^1^, ^2^, ^3^, ^4^. The elderly have a worse performance in low redundancy hearing tasks, in dichotic hearing tasks and in temporal pattern tasks when compared to adult individuals and such findings could indicate impairment in central auditory pathways^1^, ^5^, ^6^, ^7^, ^8^, ^9^, ^10^, ^11^. Right ear advantage (REA) seen in dichotic listening tests, was considered as stemming from an impairment of the inter-hemispheric auditory pathways.^8^, ^9^, ^10^, ^11^

Auditory processing assessment information is important to identify functional deficits that could be associated to difficulties in speech understanding^12^, in audiologic rehabilitation/training^13^ and to check the results of treatment interventions^14^.

Ipsilateral synthetic phrases identification test with competitive message (SSI/MCI), frequency patterns test (PPS) and the alternate dissyllable by dichotic task test (SSW) are behavioral sensitive tests, with specificity, reliability and facility to use in certain lesions or dysfunctions of the different areas of the auditory central nervous system (ACNS).

The ipsilateral synthetic phrases identification test with competitive message (SSI/MCI) proposes a monoaural task that reduces speech redundancy signal by means of speech competition, identifying brain stem involvement^5^, ^6^, ^7^, ^15^. Depending on lesion location in the brain stem, we can find the following results in the SSI/MCI:

1) ipsilateral deficit when there is lesion in the lower portion of the pons;
2) contralateral deficit when there is a lesion in the upper portion of the pons; and
3) bilateral deficit when the lesion is large and invasive^7^.

The frequency pattern test (PPS) assesses the temporal processing of auditory information, identifying the involvement of the right hemisphere (RH), left hemisphere (LH) and the inter-hemispheric conections^16^, ^17^, ^18^, ^19^, ^20^, ^21^. In PPS there are response patterns that locate the lesion in the central auditory system:

1) deficit in both ears in the murmuring and naming response modes, when there is RH involvement;
2) results within normal ranges in both ears of the murmuring type of response, associated with a deficit in both ears in the naming response mode, when there is LH involvement; and
3) results within normal ranges in both ears in the murmuring response mode, associated with deficits in both ears in the naming response mode, when there is involvement of the corpus callosum.^11^, ^17^, ^19^, ^20^, ^22^, ^23^

The alternate dissyllable test by means of dichotic tasks (SSW), which is based on the simultaneous presentation of different sounds to the right ear (RE) and the left ear (LE), identifies involvement of the brain stem, RH, LH and inter-hemisphere connections^6^, ^8^, ^24^, ^25^, ^26^. In SSW - dichotic presentations, there may be the following results:

1) deficit in the competitive right or bilateral, when there is LH involvement;
2) deficit on the competitive left, when there is RH involvement; and
3) deficit on the competitive left, when there is involvement of the corpus callosum.^6^, ^8^, ^25^

The goal of the present investigation is to assess the efficiency of central auditory function of elderly patients who report good hearing.

## MATERIALS AND METHODS

This study, approved by the Ethics Committee of the Hospital São Paulo/ Universidade Federal de São Paulo - Escola Paulista de Medicina, was carried out by the Neurotology Course, at the Department of Otorhinolaryngology-Head and Neck Surgery of this University, and approved under protocol # 258/00. The participants signed a Free and Informed Consent Form before being submitted to the proposed evaluation.

We assessed 40 patients in the age range of 60 to 75 years of age, without history of neurological disorders, right-handed, who reported good hearing. The patients were submitted to auditory processing evaluation by means of an interview; otorhinolaryngological exam; threshold tonal audiometry; speech recognition threshold (SRT); speech recognition index (SRI); immittance test; stapes reflex test; SSI/MCI, PPS and SSW, considering the response in the conditions of the competitive right (CR) and competitive left (CL), to evaluate their performance in dichotic speech tasks.

The reference used for the analyses of results was the normality pattern of adult individuals. The expected percentage of correct answers in both ears at SSI/MCI in the competition/stimulus ratio 0dB is of 80.0%, in the competition/stimulus ratio -10dB is 70%, and in the competition/stimulus ratio -15dB is 60.0%27; at SSW in the CR and CL is 90.0% of correct answers^28^; and in PPS the expected result in the naming mode is of 76% of correct answers^29^, without significant differences between the percentage of right answers in the naming and murmuring response modes^11^.

We used the t-paired and the variance analysis for the statistical study. In order to analyze the influence of gender, the individuals were broken down in two groups, one of females and one of males; regarding the age variable, the individuals were broken down into three groups, one of people between 60 and 65 years of age, another between 66 and 70 years of age and a last one, of people between 71 and 75 years of age; and the hearing loss variable, where the individuals were distributed in two groups, one with threshold averages in the frequencies of 500; 1,000; 2,000 and 3,000 Hz up to 25dB HL and in the frequencies of 4,000; 6,000 and 8,000 Hz lower than 30dB HL and another with average threshold averages in the frequencies of 4,000; 6,000 and 8,000 Hz higher than 30dB HL.

## RESULTS

Of the 40 elderly examined, 29 were women and 11 were men; 15 were between 60 and 65 years, 12 were between 66 and 70 years and 13 had between 71 and 75 years; mean age was 68.2 years, with a standard deviation of 4.4.

The descriptive values regarding tonal thresholds, speech recognition thresholds in dB HLNA, and speech recognition index are shown on Table 1.

**Table 1:** Descriptive measures associated to tonal and speech recognition thresholds in dB HL, and the speech recognition index.

Tonal thresholds, speech recognition thresholds and speech recognition									
		Right ear				Left ear			
		min.	max.	mean	sd	min.	max.	mean	sd
	0,25	5,0	25,0	15,5	4,9	5,0	30,0	16,1	5,9
	0,50	10,0	30,0	16,7	5,9	5,0	30,0	16,0	6,9
	1,0	5,0	35,0	17,5	7,5	0,0	35,0	16,1	8,5
F	2,0	0,0	45,0	19,8	11,2	5,0	50,0	19,5	11,3
(KHz)	3,0	0,0	60,0	22,1	14,5	5,0	60,0	23,7	14,6
	4,0	5,0	65,0	27,3	16,0	5,0	70,0	30,1	17,7
	6,0	0,0	90,0	36,2	20,3	5,0	90,0	39,1	21,4
	8,0	5,0	95,0	38,7	21,0	5,0	95,0	40,0	23,4
SRT (dB NA)		10,0	35,0	20,7	6,9	10,0	35,0	20,4	6,0
SRI (%)		80,0	100,0	94,0	6,0	80,0	100,0	94,0	6,4

Legend: F = frequency; SRT = speech recognition threshold; SRI = speech recognition index; min = minimum; max. = maximum; sd = standard deviation

Audiometry led to the identification of thresholds within normal ranges in all frequencies tested in eight cases (20.0%); symmetrical sensorineural hearing loss with descending shape in 24 (60.0%); and hearing loss in isolated frequencies in 8 (20.0%). Speech recognition thresholds (SRT) below or equal 25dB were found in 32 cases (80.0%) in the right ear and in 34 (85.0%) in the left ear. Percentage speech recognition indices with correct answer percentages higher than or equal to 88% were found in 35 cases (87.5%) in the right ear and in 33 (82.5%) in the left ear.

Regarding immittance testing, all the individuals presented type A curves, with contra and ipsilateral stapes reflex thresholds matching their auditory thresholds.

In the SSI/MCI we found the following results in comparison with normal values for adults: 21 individuals (52.5%) had values considered within normal ranges; ten (25.0%) had results below those of normal ranges, bilaterally; and nine (22.5%) had values which were unilaterally below normal values.

Table 2 shows the descriptive values of the SSI/MCI test according to the stimulus/competition ratio.

**Table 2:** Descriptive measures of the synthetic phrases identification test with ipsilateral competitive message - SSI/MCI according to the stimulus/competition ratio.

Synthetic phrases identification test with ipsilateral competitive message - SSI/MCI						
	Stimulus/competition ratio					
	0 dB		-10 dB		-15 dB	
Measures	Right ear	Left ear	Right ear	Left ear	Right ear	Left ear
Mean	80,5	81,7	63,2	66,0	52,5	59,7
Standard deviation	21,3	21,0	25,0	24,9	22,6	25,0
Minimum	0,0	0,0	0,0	0,0	0,0	0,0
Maximum	100,0	100,0	100,0	100,0	100,0	100,0

The statistical analysis regarding the comparison among genders, age ranges, hearing loss and right ear performance in comparison with the left ear indicated: 1) no gender influence on the results; 2) significant influence of the age range (p=0.043) on SSI/MCI results regarding the stimulus/competition ratio -15dB, with individuals between 70 and 75 years of age presenting the worse results when compared to individuals in the age range of 60 to 65 years of age; 3) significant influence of the hearing loss in the ratio -15dB (p=0.012 in the RE and p=0.004 in the LE), in the -10dB (p=0.042 in the LE) ratio, and in the 0dB (p = 0.042 in the RE and p=0.017 in the LE) ratio; and 4) significant difference between the ears (p=0.002) in the -15 dB stimulus/competition ratio, with better results associated with the LE.

In the frequency patterns test - PPS we found the following results in comparison with normal values for adults: 29 individuals (72.5%) had values within normal levels of normality in the murmuring response mode; and 20 (50.0%) presented values that were within normal ranges for the naming response mode.

Table 3 presents the descriptive measures of the frequency patterns test - PPS according to the response mode.

**Table 3:** Descriptive measures of the frequency patterns test - PPS according to the response mode.

Frequency patterns test									
		Right ear				Left ear			
Modes		mean	sd	min	max	mean	sd	min	max
murmuring	Correct	86,5	20,6	7,0	100,0	88,0	21,3	7,0	100,0
	inversions	1,7	3,5	0,0	15,0	2,6	5,2	0,0	23,0
Naming	Correct	66,8	27,7	7,0	100,0	67,9	26,4	7,0	100,0
	inversions	9,7	10,6	0,0	50,0	9,9	9,2	0,0	47,0

Legend: murm. = murmuring; nam. = naming; sd = standard deviation; min. = minimum; max. = maximum

The statistical analysis considering the naming/murmuring response mode, gender, age range, hearing loss and right ear performance in relation to the left ear indicated that:

1) significant difference between the percentage of correct answers in the murmuring response mode and the percentage of correct answers in the naming response mode, bilaterally, (p<0.001), with a higher percentage of correct answers in the murmuring response mode;
2) bilateral significant difference between the percentage of inversions in the murmuring response mode and the percentage of inversions in the naming response mode, (p<0.001), with a lower percentage of inversions in the murmuring response mode;
3) significant gender influence in both ears on the naming response mode (p=0.018), with women having more difficulties;
4) no hearing loss and age influence on the results; and
5) no significant difference between the ears.

In the alternate disyllables test - SSW we found the following results in comparison with normal values for adults: 20 individuals (50.0%) had values within normal ranges in the competition condition, bilaterally; three (7.5%) had values below normal patterns, bilaterally; 14 (35.5%) had values below normal patters in the left competitive condition; and three (7.5%) had results below normal standards in the competitive condition.

Table 4 presents the alternate disyllables test - SSW descriptive values according to the hearing condition.

**Table 4:** Descriptive measures of the alternate dissyllable test - SSW according to the hearing conditions.

Alternate dissyllable test - SSW				
	Descriptive measures			
Hearing conditions	mean	Standard deviation	minimum	maximum
Competitive right	88,1	10,1	40,0	97,0
Competitive left	81,6	13,4	45,0	97,0

The statistical analysis in relation to the comparison of gender, age, hearing loss and right ear performance in relation to the left ear showed that:

1) hearing loss, gender and age range did not influence the results in the competitive right and left conditions; and
2) there was a significant difference between the percentage of correct answers in the right and left competitive conditions (p=0.002), and the highest average of correct answers percentage was presented in the right competitive condition.

In the joint analysis of SSI/MCI, PPS and SSW we found the following results in comparison with normal values for adults: eight (20.0%) individuals presented normal results in all the tests; six (15.0%) presented low results in all the tests; 13 (32.5%) presented low results in two tests; and 13 (32.5%) presented low results in one test only.

## DISCUSSION

Obtaining information on auditory processing depends on tests which are sensitive enough to detect the involvement of different regions of the auditory system.

The patients presented hearing thresholds within normal ranges in all the frequencies tested; symmetrical and descending sensorineural hearing loss curves, and hearing loss in isolated frequencies. Most patients had SRI values within normal ranges, and this justified why these patients reported good hearing.

In the SSI/MCI test, the average correct answers percentage, in the three competition conditions (0, -10 and -15dB) was below the results considered normal for adult individuals^27^. The greatest number of errors could be interpreted as a hint of alterations in the central auditory pathways^30^ and more specifically identifying a reduction in brain stem efficacy, considering that this test is sensitive enough to detect involvement of this region of the auditory system^6^, ^7^, ^15^. Individuals in the age range between 71 and 75 years presented a higher number of errors when compared to those in the range of 60 and 65 years. There was a trend towards a larger number of errors in the age range of 66 to 70 years in comparison to patients between 60 and 65 years. These findings could suggest a slow and progressive involvement of the brain stem auditory pathways with aging. However, the significant influence of the hearing loss, which also increased with aging, prevents definitive conclusions in these regards and stresses that this test must be used carefully in individuals with presbycusis. In eight individuals with presbycusis and bilateral SSI/MCI alteration it was not possible to establish involvement of the acoustic central nervous system^31^.

In the frequency patterns test - PPS there are response patterns which are characteristic according to the lesion location in the central auditory system:

1) bilateral deficit in the murmuring and naming response modes when there is RH involvement;
2) normal results bilaterally in the murmuring response mode, associated with a bilateral deficit in the naming response mode when there is LH involvement; and
3) normal results bilaterally in the murmuring response mode, associated with a bilateral deficit in the naming response mode when there is involvement of the inter-hemisphere conections^11^, ^17^, ^19^, ^20^, ^22^, ^23^.

In the PPS test, the prevalence of results below normal ranges for adults in the naming response mode suggests that the elderly we evaluated would have impairments in the auditory pathways associated with this task, without, however, having involvement of the auditory pathways associated with the murmuring response mode^11^.

We have seen that the average of correct answers in the naming response mode for males is within normal values, and this was not seen for women. These PPS results suggest that in elderly women there is impairment of the auditory pathways responsible for naming frequency patterns^11^. Age range and hearing loss did not interfere in the analysis of results, stressing the usefulness of PPS in the elderly, regardless of hearing loss. On the other hand, the significant difference between female and male performance indicates the need to establish normality patterns according to gender.

In the SSW test, the percentages of correct answers in the CR and CL conditions show results below normal standards for adults^28^, suggesting impairment of the RH, LH and corpus callosum auditory pathway impairments by the dichotic hearing task^6^, ^8^, ^24^, ^25^. The SSW test was sensitive and efficient in the analysis of central auditory pathways in the elderly, and it is not impacted by age range, gender and hearing loss.

Based on the findings of these three tests and considering normality patterns for adult individuals, 20.0% of the elderly individuals had ACNS involvement stemming from aging. The fall in binaural processing is frequently associated with aging; however, it does not manifest itself in all cases^32^.

Altered results in all three tests, present in 15.0% of the cases, would indicate auditory pathway involvement that go from the brain stem to the cortex, including inter-hemisphere pathways. In this group, the brain stem impairment was not confirmed in three individuals, because they had hearing loss. Aging-related alterations along the entire ACNS can be associated with a widespread loss of neurons^31^.

Alterations in two tests were seen in 32.5% of the patients. Eight patients had results that suggest the involvement of cortical areas, associated with brain stem impairment; in four of these cases the brain stem involvement was not confirmed because of the hearing loss. One case had signs suggesting brain stem impairment; one case had corpus callosum impairment; one case had corpus callosum impairment and LH impairment; one case had RH impairment; one case had RH and LH impairment.

Altered results in one test only were seen in 32.5% of the elderly evaluated. In SSI, four cases were altered, and in three of them the findings suggest brain stem involvement, and in one case with hearing loss, the result was not conclusive. In the PPS we had four cases with alterations - the findings point towards a possible involvement of cortical areas, but one was not confirmed by the SSW. As far as SSW is concerned, five cases had alterations, four had a right ear advantage and one had left ear advantage, suggesting a possible brain stem involvement or that of cortical and/or corpus callosum; these possibilities were not corroborated by the SSI/MCI, as to brain stem involvement, or PPS application as far as cortical and/or corpus callosum involvement is concerned.

We have raised some hypothesis that could explain our findings:

1) ACNS structures would be affected at different times, in stages, and the individuals who did not have alterations in all the tests could present an involvement of the most external ACNS structures in future evaluations;
2) aging would affect certain ACNS structures, depending on the susceptibility of each individual;
3) the involvement observed would not happen because of aging only, it could stem from health problems along the person”s life; and
4) the group of tests chosen could not be sensitive enough to detect mild dysfunctions, despite being proven sensitive to detect ACNS lesions. Nonetheless, we did not see a relationship between general physiological factors, life style and a greater incidence of central auditory function involvement in the elderly^33^.

In our study, the analysis of the three tests together suggest that there are no response patterns that happen only with the elderly, and this has also been seen in other studies carried out with individuals of this age range^23^, ^34^. The prevalence of central auditory function involvement in the elderly and the impact it causes on their quality of hearing suggest the importance of including a central auditory evaluation in their audiologic protocol^35^. The utilization of these tests in assessing the elderly help us see impaired central auditory functions, improving the quality of therapeutic-rehabilitative interventions.

The majority (80.0%) of the elderly individuals we examined, despite having reported good hearing, presented altered results in their central auditory functions, suggesting that this is a study field that needs to be explored in future studies, with the goal of establishing the ACNS”s role in the auditory behavior of the elderly and their diagnostic and therapeutic implications.

## CONCLUSION

The prevalence of abnormalities, alone or combined, in the identification of synthetic phrases with ipsilateral competitive message, in the naming of frequency patterns and in dichotic hearing tasks is relevant and characterize impairment of the central auditory function in elderly people who report good hearing.
